# Mass spectrometry data from proteomic analysis of human skin keratins after exposure to UV radiation

**DOI:** 10.1016/j.dib.2016.02.008

**Published:** 2016-02-10

**Authors:** Seon Hwa Lee, Keita Matsushima, Kohei Miyamoto, Tomoyuki Oe

**Affiliations:** Department of Bio-analytical Chemistry, Graduate School of Pharmaceutical Sciences, Tohoku University, Sendai, Miyagi 980-8578, Japan

**Keywords:** Human skin keratins, Methionine oxidation, UV irradiation, Proteomics, LC/ESI-MS

## Abstract

A mass spectrometry (MS)-based proteomic methodology was employed to monitor oxidative modifications in keratins, the main constituents of human skin (“Non-invasive proteomic analysis of human skin keratins: screening of methionine oxidation in keratins by mass spectrometry” [1], “UV irradiation-induced methionine oxidation in human skin keratins: mass spectrometry-based non-invasive proteomic analysis” [2]). Human skin proteins were obtained non-invasively by tape stripping and solubilized in sodium dodecyl sulfate (SDS) buffer, followed by purification and digestion using the filter-aided sample preparation method. The tryptic peptides were then analyzed by liquid chromatography (LC)/electrospray ionization (ESI)-MS, tandem MS (MS/MS), and LC/ESI-selected reaction monitoring (SRM)/MS. The MS/MS data were generated to confirm amino acid sequences and oxidation sites of tryptic peptides D^290^VDGAYMTK^298^ (P1) and N^258^MQDMVEDYR^267^ (P2), which contain the most susceptible oxidation sites (Met^259^, Met^262^, and Met^296^ in K1 keratin) upon UVA irradiation [2]. Subsequently, quantitative determination of the relative oxidation levels of P1 and P1 [2] was achieved by LC/ESI-SRM/MS analyses of P1 and P2 together with their oxidized forms after exposure to UVA radiation or treatment with hydrogen peroxide (H_2_O_2_).

## 1 **Specifications table**

**Table:** 

Subject area	*Toxicology and pharmacological science*
More specific subject area	*Proteomics*
Type of data	*MS/MS spectra, LC/ESI-SRM/MS chromatograms*
How data was acquired	*Mass spectrometry using a API2000 triple quadrupole mass spectrometer (ABsciex, Framingham, MA)*
Data format	*Analyzed by Analyst*® *Version 1.5.2.*
Experimental factors	*Human skin proteins were obtained non-invasively by tape stripping and solubilized in SDS buffer, followed by purification and digestion using the filter-aided sample preparation method.*
Experimental features	*Human skin tryptic peptides were analyzed by LC/ESI-MS, MS/MS, and LC/ESI-SRM/MS.*
Data source location	*Department of Bio-analytical Chemistry,*
*Graduate School of Pharmaceutical Sciences, Tohoku University,*
*Sendai, Miyagi 980-578, Japan*
Data accessibility	*Data is with this article.*

## 2 Value of the data

• The MS/MS data confirmed the identities of tryptic keratin peptides carrying the most susceptible oxidation sites.
• The SRM/MS data revealed a time-dependent increase in the relative oxidation levels of target keratin peptides upon UVA irradiation and H_2_O_2_ treatment.
• Difference in the oxidation sites between UV and H_2_O_2_ treatment was found.
• The relative oxidation levels reported could be applied to the assessment of oxidative stress levels in skin after exposure to sunlight.
• The relative methionine (Met) oxidation levels in keratins could be used as dosimeters of skin damage.

## 3 Data

**Fig. 1:**
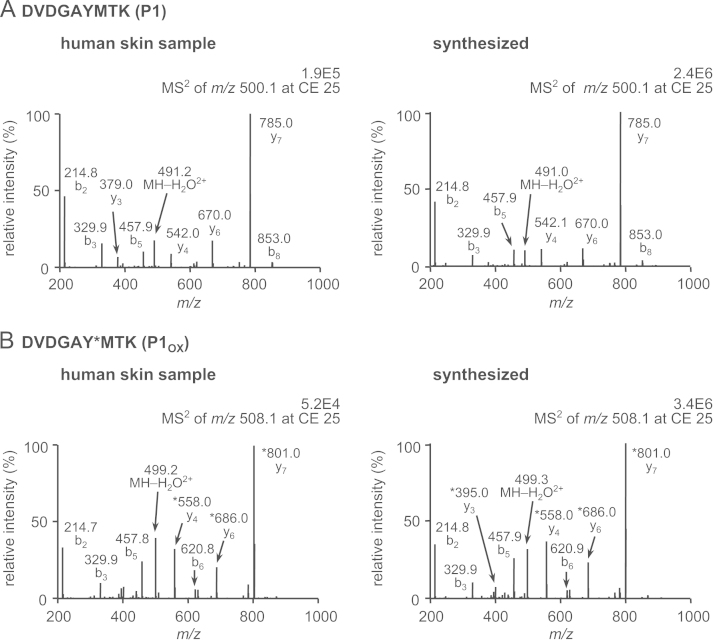

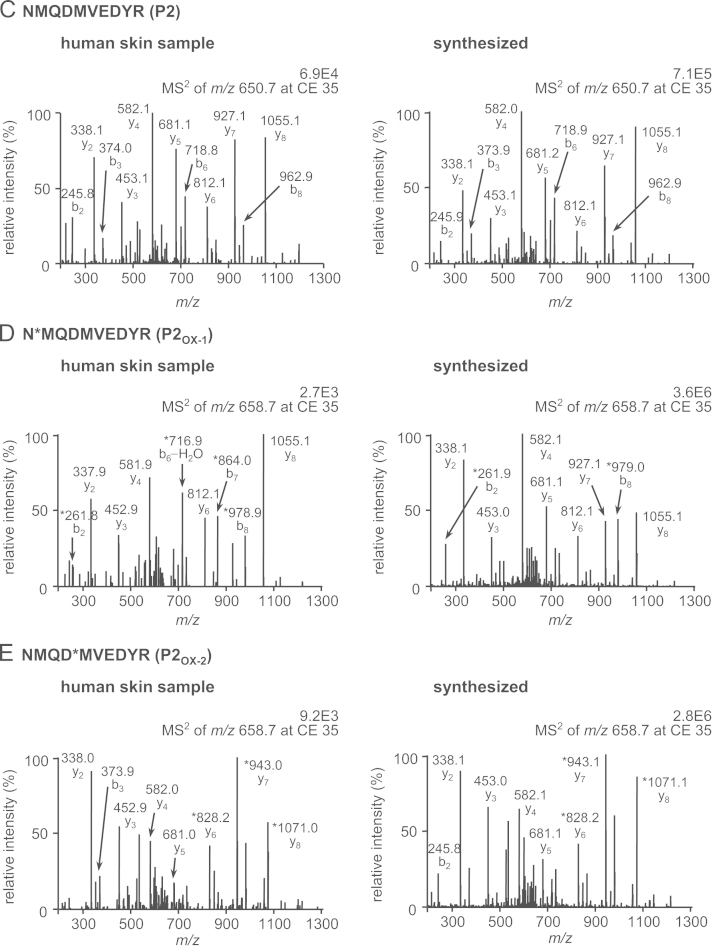
MS/MS analyses of target peptides in human skin sample (left) and synthetic peptides (right). (A) DVDGAYMTK (P1), (B) DVDGAY^⁎^MTK (P1_OX_), (C) NMQDMVEDYR (P2), (D) N^⁎^MQDMVEDYR (P2_OX-1_), and (E) NMQD^⁎^MVEDYR (P2_OX-2_). ^⁎^ Represents an oxidized site (+16 Da).

**Fig. 2:**
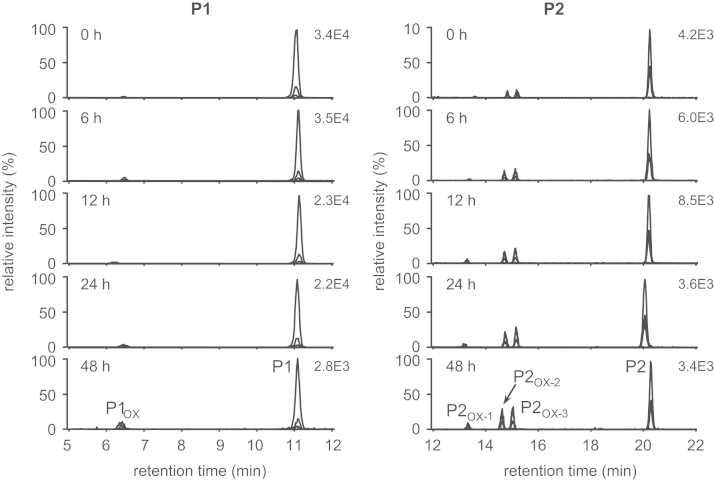
LC/ESI-SRM/MS analyses of the tryptic human skin peptides DVDGAYMT (P1, left) and NMQDMVEDYR (P2, right) with their oxidized forms after exposure to UVA for 0, 6, 12, 24, and 48 h.

**Fig. 3:**
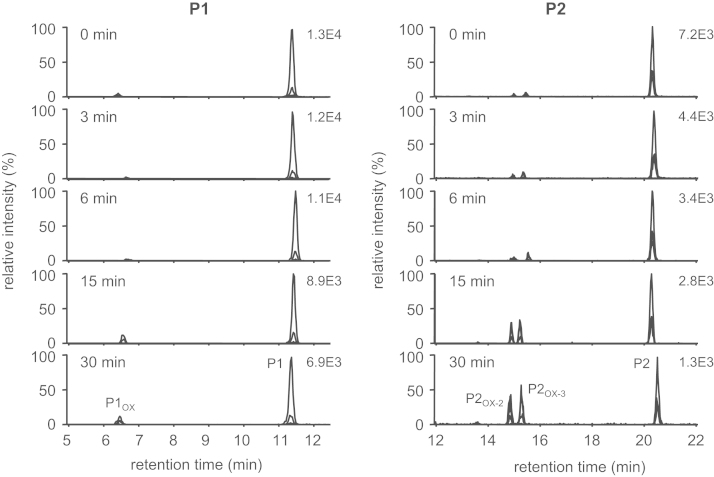
LC/ESI-SRM/MS analyses of the tryptic human skin peptides DVDGAYMT (P1, left) and NMQDMVEDYR (P2, right) with their oxidized forms after treatment with H_2_O_2_ (100 mM) for 0, 3, 6, 15, and 30 min.

Matrix-assisted laser desorption ionization/time of flight-MS analyses of UVA-irradiated human skin proteins revealed Met-containing tryptic peptides, D^290^VDGAYMTK^298^ (P1) and N^258^MQDMVEDYR^267^ (P2), as potential biomarkers of oxidative skin damage [2]. Amino acid sequences and oxidation sites of P1 and P2 were confirmed by the MS/MS analysis and comparisons to their authentic standards (Fig. 1). The UV-induced oxidative susceptibility of peptides P1 and P2 was assessed via irradiating human tape-stripped skin proteins with UVA for 0–48 h, followed by LC/ESI-SRM/MS analysis (Fig. 2). The extent of UVA-mediated oxidation of target peptides P1 and P2 was compared with that induced by H_2_O_2_ (Fig. 3).

## 4 Experimental design, materials and methods

### 4.1 Isolation and solubilization of human skin proteins

Human skin proteins were obtained by tape stripping the outermost layer of the skin with adhesive skin tapes. The tape was attached to the upper arm, gently pressed three times with a finger, and detached. To avoid contamination by surface lipids, the upper arm skin was cleaned with ethanol before the procedure and the first tape strip was discarded. After the second tape stripping, one-fourth (6.25×25 mm^2^) of the tape was placed into a 2 mL microcentrifuge tube containing 200 μL of SDS buffer (0.1% SDS, 0.15 M NaCl, and 2 mM butylated hydroxytoluene (BHT) in 50 mM sodium phosphate buffer, pH 7.0) with the adhesive side facing inward. Human skin proteins on the gluey surface of the skin tape were then solubilized in the buffer by scraping them for 4 min using a grinding plastic pestle followed by sonication for 1 min. The sonicated human skin protein mixture was centrifuged at 11,300 g for 10 min at 4 °C.

### 4.2 Preparation of human skin protein samples for MS analysis

An aliquot (150 μL) of human skin protein mixture was incubated at 54 °C for 1 h with 25 μL of dithiothreitol (20 mM) in reduction and carboxymethylation (RCM) buffer (8 M urea, 50 mM Tris–HCl, 0.1 M mercaptoethanol, 2 mM BHT, pH 8.6). The incubation mixture was transferred into a 30 K filtration unit and centrifuged for 12 min at 11,300 g. The mixture remaining in the filter unit was diluted with 100 μL of RCM buffer and centrifuged (11,300 g for 12 min). After centrifugation, the mixture was diluted with 100 μL of RCM buffer, incubated in darkness at room temperature for 45 min with 25 μL of iodoacetamide (110 mM) in RCM buffer, and centrifuged for 12 min. The mixture in the filter unit was diluted with 100 μL of 100 mM NH_4_HCO_3_ containing 2 mM BHT and centrifuged for 12 min at 11,300 g. This step was repeated three times. The filter unit containing human skin protein mixture was transferred to a new collection tube and added to 100 μL of 100 mM NH_4_HCO_3_ containing 2 mM BHT and 0.75 μg of trypsin. Following overnight incubation at 37 °C, peptides were collected by centrifugation of the filter unit for 12 min at 11,300 g. The filtrate was evaporated to dryness under a nitrogen stream and redissolved in 30 μL of 5% aqueous acetonitrile for LC/ESI-MS analysis.

### 4.3 Exposure of isolated human skin proteins to UV

Human skin proteins were obtained by tape stripping the outermost layer of upper arm skin as described in Section 4.1. Proteins on half (12.5×25 mm^2^) of the second tape were irradiated with UVA or UVB for 0, 6, 12, 24, and 48 h. UV irradiation was performed using a Dermalight 80 equipped with a UVA lamp (PL-S 9W UV-A/2P 1CT) at a dose rate of 4.5 mW/cm^2^, or with a UVB lamp (PL-S 9W/12/2P 1CT) at a dose rate of 2.0 mW/cm^2^. The UVA and UVB lamps delivered UV light in the range of 315−380 nm and 290−315 nm, respectively. After irradiation, the tapes were placed in darkness until all irradiation exposure times were complete. The irradiated tapes were cut into two pieces (6.25×25 mm^2^ each) and one piece was processed for LC/ESI-MS analysis as described in Section 4.2.

### 4.4 Treatment of isolated human skin proteins by H_2_O_2_

One-fourth of the second tape was placed into a 2 mL microcentrifuge tube containing 1.8 mL of H_2_O_2_ solution (100 mM). After incubation at 37 °C for 3, 6, 15, and 30 min, H_2_O_2_ solution was discarded and the tape was washed with water (1 mL×2). Human skin proteins on the tape were then solubilized in 200 μL of SDS buffer and processed for LC/ESI-MS analysis as described in Section 4.2.

### 4.5 LC/ESI-MS, MS/MS and SRM/MS analyses

Chromatography for LC/MS analyses were carried out using 0.1% (v/v) formic acid in water and 0.1% (v/v) formic acid in acetonitrile as solvent A and solvent B, respectively. An Agilent 1100 LC system (Agilent Technologies, Inc., Santa Clara, CA) equipped with binary pump (G1312A), autosampler (G1367A), column oven (G1316A), photodiode array detector (G1314A), and degasser (G1379A) was used. Separations were performed at 40 °C using a Jupiter Proteo column (150×2.0 mm i.d., 4 μm, 90 Å Phenomenex, Torrance, CA). The linear gradient was as follows: 5% B at 0 min, 20% B at 25 min, 95% B at 26 min, 95% B at 30 min, 5% B at 31 min, and 5% B at 45 min with a flow rate of 0.2 mL/min.

An API2000 triple quadrupole mass spectrometer (ABsciex, Framingham, MA) equipped with an ESI source was used in positive ion mode using LC system 1. The API2000 operating conditions were as follows: curtain gas, 30 psi; collision gas, 7 psi; ion spray voltage, 4500 V; source temperature, 450 °C; ion source gas 1, 50 psi; and ion source gas 2, 70 psi. Full scanning analyses were performed in the range of *m/z* 400−1400. MS/MS and collision-induced dissociation was performed using nitrogen. SRM/MS parameters are shown in Table 1 in Ref. [2]. Data analysis was performed using Analyst® Version 1.5.2.
