# Supplementary material for: Mass spectrometry data from proteomic analysis of human skin keratins after exposure to UV radiation
Source: Data Brief. 2016 Feb 10;7:100–6. doi: 10.1016/j.dib.2016.02.008 (PMC4764772; doi:10.1016/j.dib.2016.02.008)
Supplement: Supplementary file 1 — Supplementary material [file mmc1.docx]

*Conflict of interest*

**Title:** Mass spectrometry data from proteomic analysis of human skin keratins after exposure to UV radiation

**Authors:** Seon Hwa Lee*, Keita Matsushima, Kohei Miyamoto and Tomoyuki Oe*

**Affiliations:** Department of Bio-analytical Chemistry, Graduate School of Pharmaceutical Sciences,

Tohoku University, Sendai, Miyagi 980-8578, Japan

**Contact email:** sh-lee@mail.pharm.tohoku.ac.jp, [t-oe@mail.pharm.tohoku.ac.jp](mailto:t-oe@mail.pharm.tohoku.ac.jp)

**Conflict of interest**

The authors declare that there are no conflicts of interest.
